# Current and future perspectives of chimeric antigen receptors against glioblastoma

**DOI:** 10.1093/immadv/ltac014

**Published:** 2022-06-01

**Authors:** Josephine Zhang, Jesús A Siller-Farfán

**Affiliations:** Department of Biology, Johns Hopkins University, Baltimore, MD, USA; St Anne’s College, University of Oxford, Oxford, UK; Sir William Dunn School of Pathology, University of Oxford, Oxford, UK

**Keywords:** glioblastoma, chimeric antigen receptors, cancer immunotherapy, solid tumors, adoptive cell transfer, adoptive cell therapy

## Abstract

Glioblastoma multiforme (GBM) is the most malignant form of cancer in the central nervous system; even with treatment, it has a 5-year survival rate of 7.2%. The adoptive cell transfer (ACT) of T cells expressing chimeric antigen receptors (CARs) has shown a remarkable success against hematological malignancies, namely leukemia and multiple myeloma. However, CAR T cell therapy against solid tumors, and more specifically GBM, is still riddled with challenges preventing its widespread adoption. Here, we first establish the obstacles in ACT against GBM, including on-target/off-tumor toxicity, antigen modulation, tumor heterogeneity, and the immunosuppressive tumor microenvironment. We then present recent preclinical and clinical studies targeting well-characterized GBM antigens, which include the interleukin-13 receptor α2 and the epidermal growth factor receptor. Afterward, we turn our attention to alternative targets in GBM, including less-explored antigens such as B7-H3 (CD276), carbonic anhydrase IX, and the GD2 ganglioside. We also discuss additional target ligands, namely CD70, and natural killer group 2 member D ligands. Finally, we present the possibilities afforded by novel CAR architectures. In particular, we examine the use of armored CARs to improve the survival and proliferation of CAR T cells. We conclude by discussing the advantages of tandem and synNotch CARs when targeting multiple GBM antigens.

## Introduction to glioblastoma and chimeric antigen receptor T cell therapy

Glioblastoma multiforme (GBM) is the most malignant and common form of cancer in the central nervous system (CNS) [1]. It is classified as a grade IV glioma and arises from astrocytes and oligodendrocytes [1, 2]. In adults, GBM primarily affects individuals over 55 years of age and has an annual incidence rate of 3.23 per 100,000 people [1, 3, 4]. Standard treatment for GBM relies on combination therapy using surgical resection, radiotherapy, and chemotherapy with temozolomide (TMZ), a DNA alkylating agent [2, 5, 6]. Even with comprehensive treatment, GBM has a poor prognosis, as the median overall survival is between 16 and 20 months and the 5-year survival rate is 7.2% [3, 4, 7]. Moreover, the standard treatment methods pose toxicities; high levels of radiation have increased risks of radionecrosis, while TMZ can cause thrombocytopenia [5, 8]. The treatments themselves can promote cancer recurrence, which occurs in 90% of GBM cases [9]. This is because both radiotherapy and TMZ are mutagens and TMZ has been associated with hypermutation, causing recurrent tumors to be more aggressive than the initial tumor [10, 11]. The severity of GBM and the inability of standard treatments to produce sustained remissions highlight the need for improved cancer therapies.

**Figure 1:**
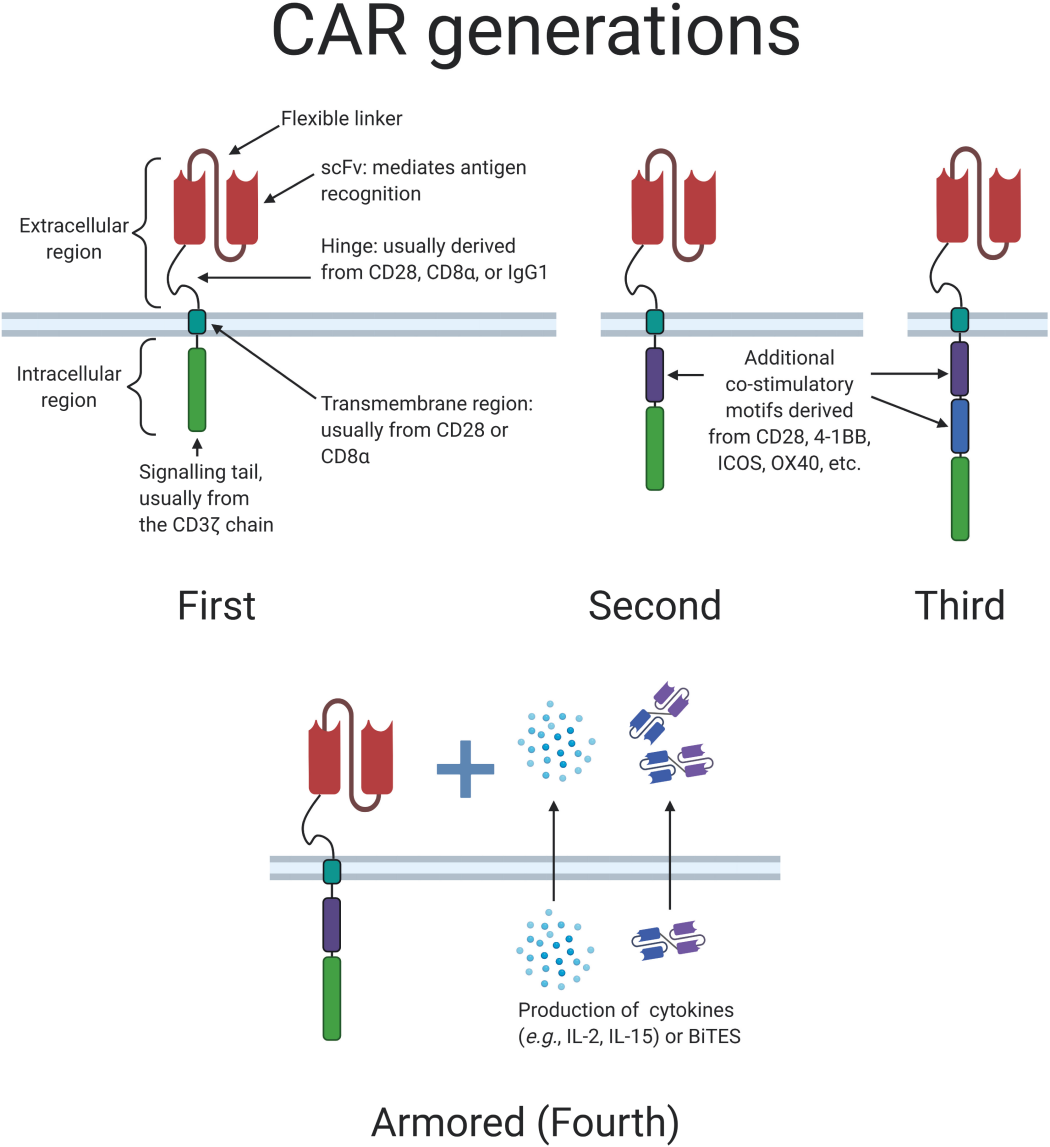
Schematic structures of chimeric antigen receptors (CARs). CARs are synthetic receptors that enable T cell recognition of diverse ligands with an antibody-like specificity. They are usually generated by fusing an antibody-derived single-chain variable fragment (scFv) with a hinge, a transmembrane domain, and a signaling tail. First-generation CARs derive their signaling from a single element, usually the zeta chain (CD247) of the T cell receptor (TCR). Second- and third-generation CARs contain one or two additional co-stimulatory motifs derived from co-receptors such as CD28, 4-1BB (CD137), or ICOS (CD278). More recently, CAR T cells have been engineered to express soluble factors in addition to CARs. These armored (or fourth-generation) CAR T cells can produce cytokines or bispecific T cell engagers (BiTEs).

One promising approach against cancer is adoptive cell transfer (ACT) using genetically engineered cells expressing chimeric antigen receptors (CARs). CARs are synthetic receptors that can be generated by fusing an antibody-derived single-chain variable fragment (scFv) with a hinge, a transmembrane domain and an intracellular signaling tail [12, 13] (Fig. 1). Unlike the native T cell receptor (TCR), CARs can target surface antigens independent of major histocompatibility complex (MHC) restriction. The intracellular tail has evolved over time; first-generation CARs contain only the CD3ζ chain (CD247) of the native TCR complex, while subsequent generations are characterized by the presence of both the CD3ζ chain and co-stimulatory domains derived from T cell co-receptors. These co-receptors include CD28, 4-1BB (CD137), ICOS (CD278), and OX40 (CD134), which can improve proliferation and survival [12, 14–16]. The most recent CARs are designed to express additional products, such as cytokines or bispecific T cell engagers (BiTEs) to further enhance function [14] (Fig. 1). CAR T cells have already seen success in the treatment of hematological malignancies, as the FDA approved the first two CAR T cell therapies in 2017. Tisagenlecleucel (Kymriah) and axicatabgene ciloleucel (Yescarta) have both shown remarkable long-term efficacy for the treatment of B-cell leukemia and lymphoma [17, 18]. More recently, the FDA approved three additional CAR T cell therapies: lisocabtagene maraleucel (Breyanzi) and brexucabtagene autoleucel (Tecartus) against CD19 in B-cell malignancies, and idecabtagene vicleucel (Abecma) against the B-cell maturation antigen (BCMA, also known as TNFRSF17) in myeloma [19–21].

## The challenges presented by glioblastoma

**Figure 2:**
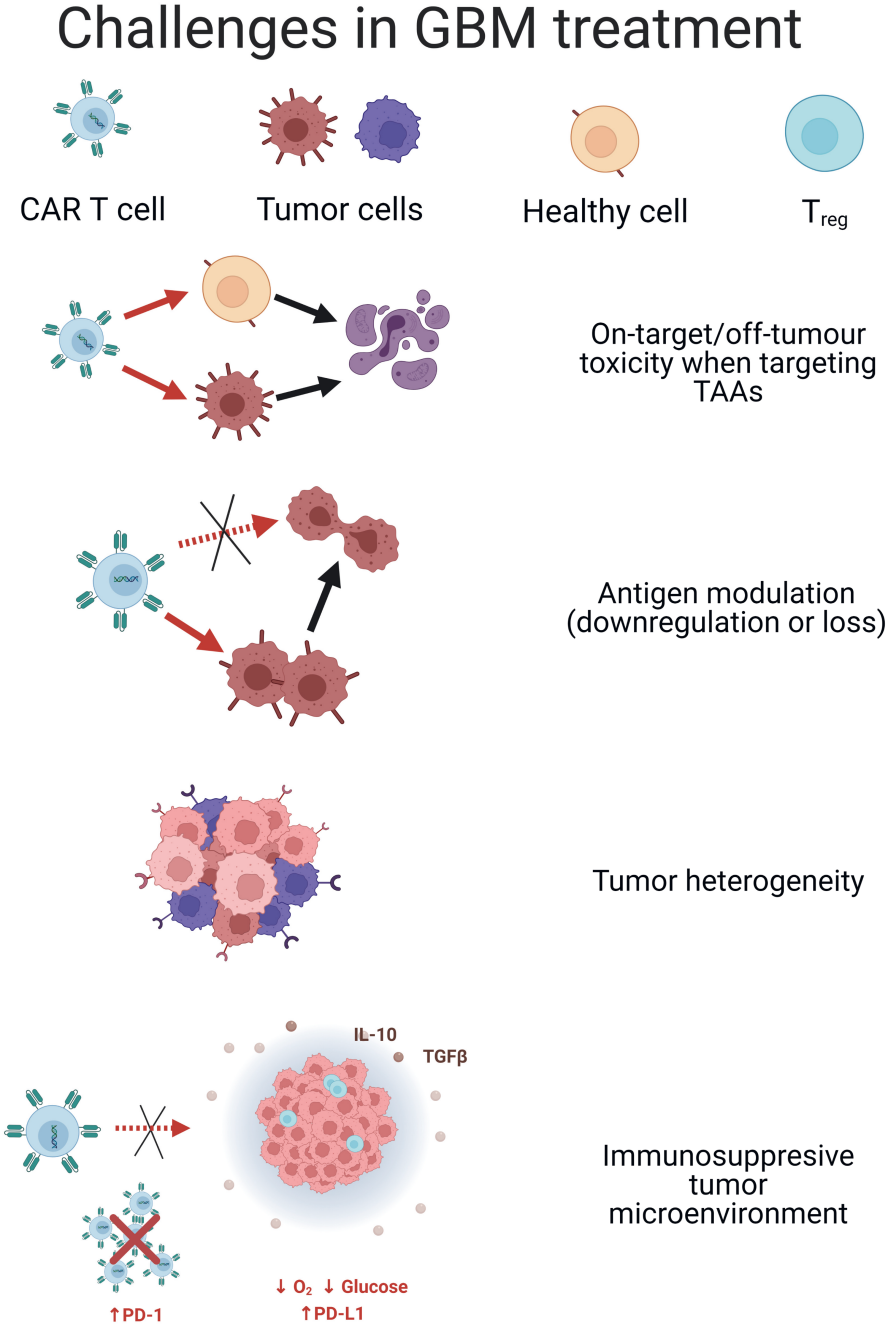
Unmet needs in ACT against GBM. CARs have shown clinical success in a number of hematological malignancies. However, there are multiple challenges limiting the efficacy of CAR T cells when targeting solid malignancies such as GBM. On target, off-tumor toxicity occurs when CAR T cells targeting a tumor-associated antigen (TAA) overexpressed by malignant cells destroy healthy tissues bearing low (physiological) levels of the same target antigen. Even if a tumor-specific antigen (TSA) is targeted by CARs, cancer cells can downregulate or lose expression of the TSA, achieving immune evasion. Other problems include the inherent heterogeneity found in GBM (which can cause different clonal lineages resistant to CARs to appear after ACT) and the immunosuppressive tumor microenvironment (TME). The TME in GBM hampers CAR homing and proliferation due to the low levels of nutrients and oxygen, as well as the presence of regulatory T cells (T_regs_) and immunomodulatory cytokines such as interleukin (IL)-10 and transforming growth factor (TGF)-β.

While CAR T cells in hematological malignancies have been efficacious, successful results have not been achieved in solid tumors, including GBM, for several reasons. Overactivation of CARs when targeting solid tumors can induce cytokine release syndrome (CRS), leading to organ failure and death [22, 23]. On-target, off-tumor effects, where CAR T cells target healthy cells expressing low levels of tumor-associated antigens (TAAs), have been observed in treating both hematological and solid malignancies. However, they are a particular concern for solid tumors, as few suitable TAAs on solid tumors have been identified [22, 24]. Two challenges relevant in GBM include antigen heterogeneity and the immunosuppressive tumor microenvironment (TME). GBM exhibits intratumoral heterogeneity, where cells in different regions of one tumor express varying mutations and evolve from separate clonal lineages. Because of this, a CAR targeted against one antigen may not be effective for the entire population of tumor cells [25]. Furthermore, heterogeneity provides a mechanism of antigen escape. This has been documented in GBM patients who lose expression of the EGFRvIII tumor-specific antigen (TSA) after treatment with anti-EGFRvIII CAR T cells [26]. Immunosuppression in the TME adds to the ability of GBM to evade CAR T cell therapies, as the TME promotes an anti-inflammatory response to prevent the proliferation and persistence of T cells [12, 24, 27, 28]. Both tumor cells and surrounding cells, including microglia and tumor-associated macrophages, secrete inhibitory cytokines, upregulate expression of suppressive ligands such as programmed death-ligand 1 (PD-L1), and increase the activity of regulatory T cells (T_regs_) [27, 29, 30]. Together, the various features of GBM contribute to an unfavorable environment for CAR T cell efficacy and survival (Fig. 2).

Considering the challenges presented by GBM, both in terms of standard treatments and novel approaches, we review recent preclinical and clinical studies regarding CAR T cell applications for GBM treatment. We divide this work into four main sections: we first discuss the existing preclinical and clinical landscape of CARs targeting conventional antigens in GBM, such as the interleukin-13 receptor α2(IL13Rα2) and the epidermal growth factor receptor (EGFR). Then, we focus our attention on novel antigens against GBM, including the B7-H3 (CD276) and chondroitin sulfate proteoglycan 4 (CSPG4) molecules. Finally, we review engineering techniques that could be employed to increase the activity and persistence of these CAR T cells in the context of GBM.

## Preclinical advances in traditional CAR T cells for glioblastoma treatment

To mitigate the risk of on-target, off-tumor effects, CAR T cells must be able to target antigens highly expressed on GBM cells with minimal to no expression elsewhere. These targets are either TSAs or TAAs; TSAs arise from mutations and are characteristic of cancer cells, while TAAs are overexpressed on cancer cells but also present in low levels on other tissues [31, 32]. For GBM, the most well-characterized CAR antigens include IL13Rα2 and EGFR variant III (EGFRvIII).

IL13Rα2 is a TAA that is overexpressed by more than 60% of GBM tumors and has low levels of expression on healthy brain tissue. Its expression is associated with poor prognosis in patients [33–35]. Such features mean that IL13Rα2 is a promising CAR T cell target for GBM treatment. A study by Brown *et al*. [36] developed a second-generation 4-1BB co-stimulatory (41BB.3ζ) CAR targeting IL13Rα2 and transduced this construct into central memory T cells. In mice implanted with tumors, the IL13Rα2-targeted CAR T cells had no off-target effects or toxicities and exhibited improved survival. Compared to a first-generation IL13Rα2-targeted CAR developed by the same group, the second-generation CARs improved survival even when 10 times fewer cells were administered during treatment [36].

Additional studies [37] using this second-generation CAR construct demonstrated that CD4^+^ CAR T cells had more potent antitumor effects compared to CD8^+^ CAR T cells. Mouse xenograft models treated with a CD8^+^-only CAR T cell population saw tumor regression followed by relapse, while those administered with both CD4^+^ and CD8^+^ CAR T cells survived for over 250 days. Compared to CD8^+^ CAR T cells, CD4^+^ CAR T cells also had more robust proliferation and were able to provide protection through several rounds of GBM tumor rechallenge. When a 1:1 ratio of CD4^+^ and CD8^+^ CAR T cells were tested in an *in vitro* tumor rechallenge assay, the presence of both CD4^+^ and CD8^+^ cells did not have improved antitumor effects compared to CD4^+^ CAR T cells alone, although the population of CD4^+^ T cells did appear to increase CD8^+^ T cell proliferation [37]. While this result has not been independently established in other studies, which support synergy between CD4^+^ and CD8^+^ CAR T cells, this study does provide evidence that CD4^+^ CAR T cells are necessary to establish long-term tumor control [38, 39].

IL13Rα2-targeted CAR T cells are also able to engage endogenous immune cells and promote anti-GBM immunity. In one study conducted by Pituch *et al*. [40], immunocompetent mice receiving CAR T cell treatment were resistant to relapse in a GBM rechallenge assay, supporting previous data that CAR T cells can generate persistent protection against tumors. Moreover, in mice expressing both IL13Rα2 and a surrogate antigen (ovoalbumin, OVA), treatment with IL13Rα2-CAR T cells led to an increase in the CD8α^+^ dendritic cell population at the tumor site, followed by a CD8^+^ T cell response against OVA [40]. Given that tumor heterogeneity and antigen escape are current obstacles for treating GBM, evidence of antigen spread resulting from CAR T cells may be important for establishing immunity against these tumors [41, 42].

A second well-characterized GBM antigen is EGFRvIII, which is a TSA that is overexpressed in 31% of GBMs. It is a mutated variant of EGFR, and its expression is correlated with poor prognoses [43, 44]. Although EGFRvIII is a TSA (which precludes the risk of on-target, off-tumor toxicity when targeting this antigen), the heterogeneity of GBM poses problems of antigen escape. To prevent this, recent studies have attempted to target two GBM antigens simultaneously. In one study, a third-generation CD28.41BB.3ζ CAR construct was designed with an scFv recognizing an epitope found on both EGFRvIII and overexpressed wild-type EGFR, which occurs in 40-60% of all GBMs. In mouse xenograft models, these CAR T cells could eliminate tumor cells expressing EGFR, EGFRvIII, or both antigens, which led to prolonged survival of mice receiving treatment. Few toxicities were observed on healthy tissues expressing EGFR, indicating that the CAR T cells were specific to overexpressed EGFR and EGFRvIII [45]. A study by Choi *et al*. [46] also targeted both EGFRvIII and EGFR, but used T cells transduced with an EGFRvIII-targeted CAR construct co-expressing BiTEs against EGFR. These BiTEs bound to CD3 on T cells and EGFR simultaneously, allowing the CAR T cells to target tumor cells expressing EGFRvIII, EGFR, or both. BiTEs were also able to elicit bystander activation, directing non-transduced T cells to EGFR. By secreting BiTEs into the local tumor environment, compared to systemically administering them, off-tumor toxicities can be minimized, while still targeting heterogeneous tumors.

A third study, focusing on the targeting of EGFRvIII, administered TMZ prior to CAR T cell infusion as a form of lymphodepletion, which is thought to increase CAR T cell activity and survival [47, 48]. Specifically, dose-intensified TMZ (100 mg/m^2^/day over 21 days in a 28-day cycle) increased CAR T cell expansion in mice with EGFRvIII^+^ GBM and improved survival, compared to standard TMZ (200 mg/m^2^/day over 5 days in a 28-day cycle). Mice treated with dose-intensified TMZ before CAR T cell administration survived for a median of 174.5 days, compared to 69.5 days in mice pretreated with standard doses of TMZ [49]. Based on these findings, the combination of a standard chemotherapeutic drug such as TMZ with CAR T cell treatment may be advantageous for widespread clinical application.

## Clinical trials of CAR T cells against glioblastoma

Although CAR T cells are not yet established as a standard treatment for GBM, they are increasingly being studied in clinical trials (Table 1). Recent trials have focused on targeting GBM antigens that have been well characterized in preclinical studies, such as IL13Rα2, EGFRvIII, and human epidermal growth factor receptor 2 (HER2/neu). The trials have also assessed the safety profile of CAR T cells in patients. This work has contributed to defining the limits of CAR T cell administration, while also providing preliminary insights into how CAR T cell efficacy can be improved. In a Phase I trial (NCT01109095) using second-generation CD28.3ζ CAR T cells targeted against HER2/neu, an overexpressed TAA of GBM, no severe toxicities occurred for doses of up to 1 × 10^8^ cells. However, out of 17 patients who received CAR T cell treatment, only one had a partial response (30% reduction in the size of the largest tumor) that lasted over 9 months; seven other patients saw stable disease. The limited efficacy of the CAR T cells in this trial can be likely attributed to the lack of expansion *in vivo*. For patients receiving a single treatment dose, CAR T cells could not be detected in the peripheral blood beyond 6 weeks after the infusion. Given that the cells could persist but not proliferate, improving the proliferative capabilities of the CAR T cells may increase their antitumor effects [50].

**Table 1:** Selected completed and ongoing clinical trials of CAR T cells against GBM

Target	ClinicalTrials.gov Identifier	Phase	Intervention	Estimated sample size (*n*)	Primary outcome measures	Additional notes
EGFRvIII	NCT02664363	I	EGFRvIII CAR T cells following dose-intensified TMZ	3	MTD	Terminated (study funding ended)
EGFRvIII	NCT03726515	I	EGFRvIII CAR T cells with pembrolizumab (PD-1 inhibitor)	7	Number of subjects with treatment-related adverse effects	Completed
EGFRvIII	NCT03283631	I	EGFRvIII CAR T cells	24	MTD after intracerebral administration	Suspended
EGFRvIII	NCT01454596 [51]	I/II	EGFRvIII CAR T cells + aldesleukin following lymphodepletion (fludarabine +cyclophosphamide)	18	1. Number of treatment-related adverse events 2. PFS	No treatment-related adverse events except at highest dose level (10^10^); Median PFS = 1.3 months, one outlier of 12.5 months
EGFRvIII	NCT02209376 [26]	I	EGFRvIII CAR T cells	10	Number of adverse events	No dose-limiting toxicities; Median overall survival (OS) = 251 days
IL13Rα2	NCT04661384	I	IL13Rα2-specific hinge-optimized 4-1BB co-stimulatory CAR T cells	30	1. Incidence of adverse events 2. Overall survival (OS)	Recruiting
IL13Rα2	NCT04003649	I	IL13Rα2 CAR T cells with nivolumab or nivolumab + ipilimumab	60	1. Incidence of adverse events 2. DLT 3. Feasibility	Recruiting
IL13Rα2	NCT02208362 [52]	I	IL13Rα2-specific hinge-optimized 4-1BB co-stimulatory CAR T cells	92	1. Incidence of grade 3 toxicity 2. Incidence of DLT	Ongoing; One patient had sustained response for 7.5 months after intraventricular infusions
HER2/neu	NCT01109095 [50]	I	HER2/neu CAR CMV-specific CTLs	16	Number of subjects with DLT after CTL infusion	No toxicities up to 1 × 10^8^/m^2^, one partial response
HER2/neu	NCT03389230	I	HER2(EQ)BBζ/CD19t+ T_CM_ cells	42	1. Incidence of grade III adverse events 2. DLT	Recruiting
B7-H3 (CD276)	NCT04385173	I	B7-H3 CAR T cells between cycles of TMZ	12	1. Incidence and type of adverse events 2. MTD 3. OS and PFS	Recruiting
B7-H3 (CD276)	NCT04077866	I/II	B7-H3 CAR T cells between cycles of TMZ or TMZ alone	40	OS	Recruiting
NKG2DL	NCT04717999	NA	NKG2D CAR T cells	20	Number of participants who experience DLT	Not yet recruiting
MMP2	NCT04214392	I	CLTX (EQ)-CD28-CD3ζ-CD19t CAR T cells	36	DLT	Recruiting
CD147	NCT04045847	Early I	CD147 CAR T cells	31	Incidence and type of adverse events	Recruiting

DLT, dose-limiting toxicity; CTL, cytotoxic T lymphocyte; T_CM_, central memory T cell; MTD, maximum tolerated dose; PFS, progression-free survival; OS, overall survival; PD-1, programmed cell death protein 1.

Some clinical trials have attempted to target EGFRvIII in GBM using CAR T cells with varying success. In one Phase I/II trial (NCT01454596) [51], where third-generation CD28.41BB.3ζ CAR T cells and interleukin (IL)-2 were administered after lymphodepletion, dose-limiting toxicities did not occur in patients except for those who received dosages >10^10^ cells. One of 18 patients died during treatment (having developed grade 5 pulmonary toxicity), while another suffered a grade 3 pulmonary toxicity, which required management with steroids and continuous positive airway pressure. Although no other severe toxicities occurred, this study resulted in no objective responses, as most patients developed progressive disease. As a result, this study was terminated before reaching Phase II. Biopsies of three patients after treatment indicated the absence of EGFRvIII on recurrent tumors, suggesting that antigen escape was likely involved in limiting the success of tumor control [51]. A second clinical trial using EGFRvIII-targeted CAR T cells was a Phase I study (NCT02209376) that administered CAR T cells to 10 patients through intravenous infusion. None of the patients receiving CAR T cells experienced serious toxicities and one patient remained alive for over 18 months without additional therapy. Surgical resection of tumors after CAR T cell infusion revealed that, although the cells were able to traffic to the tumor site, there was also an influx of T_regs_ into the tumor, accompanied by the increase of immunosuppressive markers such as FOXP3 and PD-L1 [26]. These likely contributed to the limited efficacy of the CAR T cells. Overall, recent clinical trials involving EGFRvIII-targeted CAR T cells in GBM patients have proved that CAR T cells can be well tolerated in doses below 10^10^ cells, but face challenges of tumor heterogeneity and immune suppression. Both obstacles can prevent sustained antitumor responses.

A recent clinical trial (NCT02208362) using IL13Rα2-directed CAR T cells in GBM reported significant benefit in one of its patients. This patient received CAR T cells through intracranial delivery using a catheter and underwent six weekly infusions, each with a dose consisting of no more than 10^7^ cells. Tumors were initially controlled, but after lesions metastasized to the spine, 10 additional intraventricular infusions were administered. This led to a significant reduction in the size of all tumors and complete elimination of the spinal tumors, with the response being sustained for 7.5 months after the start of the treatment. However, after the final intraventricular infusion, tumors recurred at new locations. As studies of these recurring tumors indicate decreased expression of IL13Rα2, antigen escape likely contributed to continued tumor growth [52]. This study suggests that local delivery of CAR T cells and repeated infusions can improve the anticancer response. As supported by preclinical studies of CAR T cells for other CNS tumors, where local delivery yielded greater survival benefits than intravenous infusions, intraventricular infusions may have promoted CAR T cell trafficking through the cerebrospinal fluid (CSF) to target the metastatic tumors in the spine [53]. In addition, CAR T cells persisted in the CSF for at least 7 days following each infusion [52]. Especially in aggressive GBM, local CAR T cell administration with multiple doses may have positive effects on CAR T cell persistence and the durability of tumor control.

## Novel experimental techniques to combat glioblastoma

Having presented an overview of the preclinical and clinical landscape of CARs targeting conventional GBM antigens, we will now review the prospects of harnessing novel antigens when treating GBM. While studies targeting IL13Rα2 or EGFRvIII have been crucial to driving clinical trials involving CAR T cell therapy in GBM, they have also indicated that the heterogeneous antigen expression in tumor cells poses difficulties. Preclinical studies are increasingly focused on identifying additional antigens that are highly expressed in GBM, as well as incorporating alternative CAR architectures.

### Novel CAR antigens in glioblastoma

Recent work regarding CAR T cells in GBM treatment has identified antigen targets such as B7-H3 (CD276), CSPG4, carbonic anhydrase IX (CAIX), and GD2. B7-H3 is a type I transmembrane protein that is highly expressed in over 70% of GBMs and has low expression in healthy brain tissue [54–56]. In one study using second-generation CD28.3ζ or 41BB.3ζ CAR T cells targeting B7-H3, over 86% of mice with GBM expressing B7-H3 had tumor regression and improved survival upon receiving treatment without signs of on-target, off-tumor toxicities [54]. Another study developed third-generation CD28.41BB.3ζ CAR T cells targeting B7-H3. These cells also had strong and specific cytotoxic effects in mouse models, with dose-dependent tumor regression [57]. In both cases, tumors did recur, but B7-H3 was retained, indicating that antigen loss was not involved. Some tumors likely have low levels of B7-H3 expression that prevent effective CAR T cell targeting and lead to tumor survival [54]. To reduce tumor recurrence and account for heterogeneity, future CAR constructs may need to target B7-H3 along with additional antigens.

Similar to B7-H3, CSPG4 is also a type I transmembrane protein that can be overexpressed in GBM [58–60]. One study indicated that in 67% of GBM samples assessed through immunohistochemistry, CSPG4 was highly expressed and correlated with poor overall survival. Second-generation 41BB.3ζ CAR T cells targeting this antigen were administered in mice bearing GBM xenografts and were able to eliminate tumors in 60% of the treated animals for up to 180 days. Moreover, *in vitro* studies indicated that CSPG4 expression could be upregulated by tumor necrosis factor (TNF)-α, as CSPG4 protects tumor cells from TNF-α induced apoptosis [60]. Thus, inducing upregulation of CSPG4 using TNF-α may be able to improve the antitumor activity of CSPG4-targeted CAR T cells. In a clinical context, TNF-α administration could increase toxicities, as this cytokine is involved in inflammatory responses and CRS [61, 62]. Using TNF-α in low doses, or engineering CARs that can secrete this cytokine locally, may be a viable method of supplementing CSPG4-directed CAR T cells without posing serious adverse effects.

A third novel antigen that has been identified is CAIX, a membrane-localized protein that helps maintain intracellular pH. It is highly expressed in GBM, correlated with decreased overall survival, and expressed in response to the hypoxic conditions that are common to tumors [63, 64]. In one study, third-generation CD28.41BB.3ζ CAR T cells targeting this antigen produced cytotoxic effects *in vitro* and *in vivo* against tumor cells expressing CAIX in hypoxic conditions. In 2 of 10 mice with GBM xenografts, CAR T cells achieved a sustained remission, with no tumor recurrence after 2 months. On-target, off-tumor toxicities were avoided here by using intratumoral injection, rather than systemic infusion, to administer the CAR T cells. This work also indicated that anti-angiogenic drugs that induce hypoxia, such as bevacizumab and sorafenib, can increase antitumor effects when combined with CAIX-targeted CAR T cells. These drugs are currently used to produce short-term clinical benefits in patients with recurrent GBM; if administered with CAR T cell treatment, synergistic effects between the two may be able to induce sustained tumor control [65].

GD2 is a disialoganglioside that can be expressed at high levels in GBM. Its physiological expression is restricted to peripheral nerves, neurons, and skin melanocytes, where it only represents 1–2% of the total gangliosides [66]. Using an *in vitro* co-culture with patient-derived GBM cells, Prapa *et al*. [67] demonstrated that autologous second-generation anti-GD2 CARs could successfully eradicate the malignant cells. In this same study, intracerebral (but not intravenous) administration of GD2-targeted CAR T cells mediated delayed tumor growth and improved survival in a xenograft model. Syngeneic models [68] have suggested that the combination of radiotherapy and anti-GD2 CD28.3ζ CAR T cells can improve the survival of immunocompetent mice. A recent phase I clinical trial [69] (NCT04196413) of GD2 CARs against H3K27M-mutated diffuse midline glioma did not observe instances of on-target, off-tumor toxicity, although all patients exhibited CRS (grades 1–3) that was managed with tocilizumab and corticosteroids.

### Beyond a single scFv: simultaneous antigen targeting and repurposing native receptors

**Figure 3:**
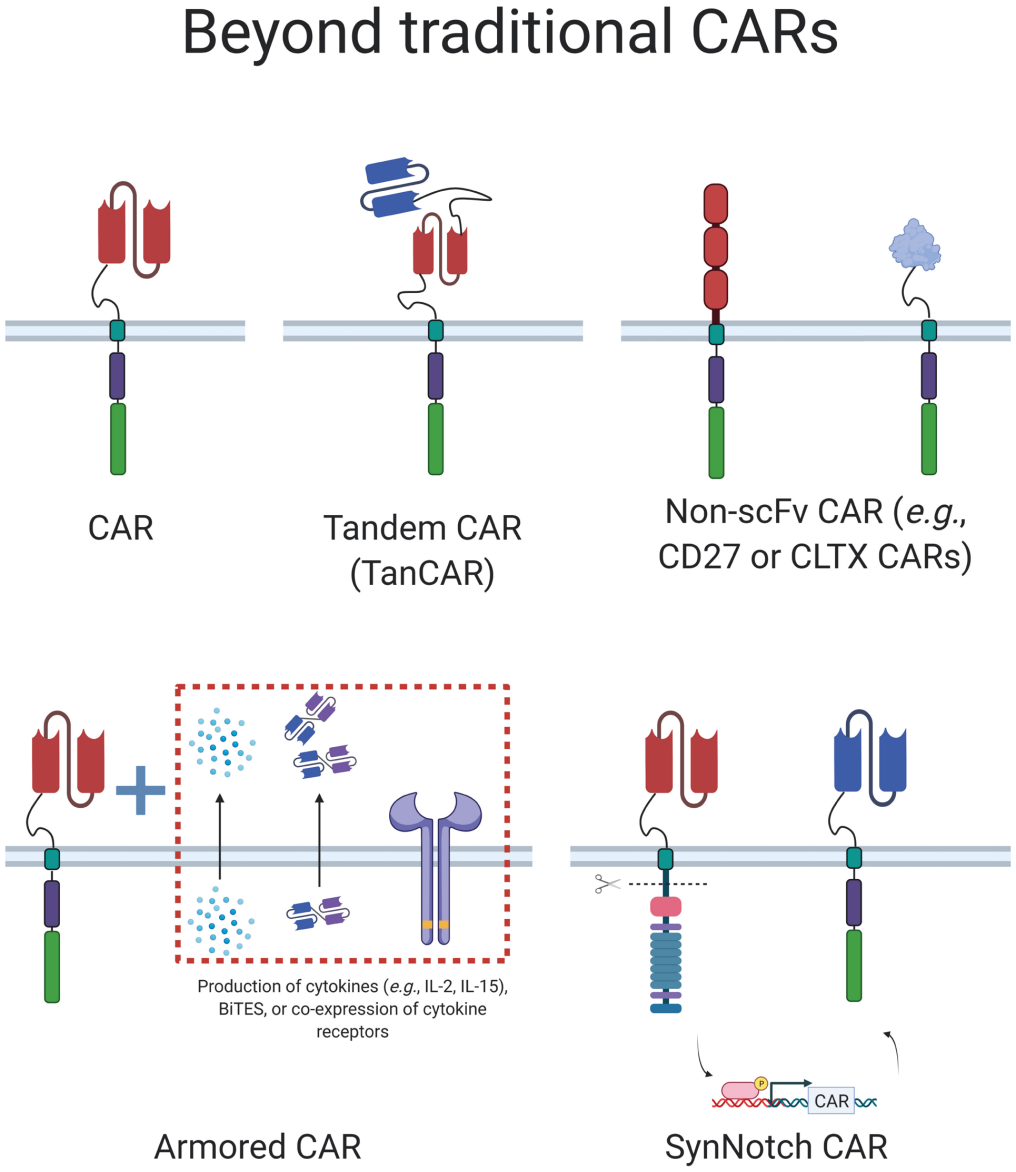
Expanding the function of GBM-directed CARs. Efforts to improve ACT against GBM can take different forms. Tandem CARs (TanCARs) can target two antigens simultaneously, something that can also be achieved by co-transduction of multiple individual CARs. Recent work has explored the possibility of generating CARs with non-scFv recognition domains. CARs using the CD27 or NKG2D ectodomains can target malignant cells expressing CD70 and NKG2DL, respectively. CARs using chlorotoxin (CLTX) as a binding motif have also been developed. Armored (or fourth generation) CARs aim to improve the effectiveness of ACT by engineering T cells so that, in addition to CARs, they express cytokines, BiTEs or cytokine receptors. Another approach is that of synNotch CARs, which repurpose elements of the Notch signalling pathway. A synNotch system can enable the controlled targeting of two antigens (AND gate), with the first CAR (red scFv) releasing the Notch intracellular domain upon antigen recognition, which in turn enables the production of a second CAR (blue scFv) recognizing a different antigen. Under this set up, the presence of both antigens is required to kill a target cell, reducing the risk of CAR T cells destroying healthy tissues.

In a study by Hegde *et al*. [70], tandem CAR T cells (TanCARs) (Fig. 3) were developed to target both HER2/neu and IL13Rα2 using a single CAR molecule. These CARs were designed based on immunofluorescence assays that indicated HER2/neu-targeted CAR T cells increased expression of IL13Rα2 and decreased expression of HER2/neu on GBM cells, whilst IL13Rα2-targeted CAR T cells produced the opposite effect. Thus, targeting both antigens could mitigate tumor escape. When assessed in mouse xenograft models in an *in vivo* stress test, TanCARs were able to increase progression-free survival compared to treatment with cells expressing individual CARs against the two antigens. Recurrent tumors were also smaller in size for mice treated with TanCARs. The ability for TanCARs to engage cells expressing either antigen, as well as those expressing both HER2 and IL13Rα2, likely contributed to their improved antitumor effects [70]. In an extension of dual-targeting CARs, a separate study developed CAR T cells targeting three antigens. These cells were transduced with constructs for three individual CARs against HER2, IL13Rα2, and ephrin-A2 (EphA2). In mouse xenograft models, these CARs increased survival and had more sustained antitumor effects compared to single and dual-targeted CAR T cells. Although tumors did recur with decreased expression of at least one of the three antigens, targeting three antigens appeared to offer significant antitumor benefits [71].

While most CAR T cells express scFv sequences derived from antibodies to target-specific antigens, CARs can be modified to express ligands to utilize receptor–ligand interactions (Fig. 3), increasing the range of possible CAR targets. One such target is CD70, a type II transmembrane protein that is typically found only on activated T and B cells, but that can be overexpressed on gliomas. In one study, RNA sequencing data indicated that high expression levels of CD70 were found on primary and recurrent GBMs, with expression being correlated with poor survival. In addition, CD70 was not present on immune cells within the tumor. This study then generated CAR T cells expressing CD27 (a TNF-family receptor that specifically binds to CD70), and transferred these cells into mice bearing tumor xenografts. Mice treated with 10^7^ CAR T cells had complete tumor regression, while treatment with 10^6^ cells led to a partial response. No toxicities were observed, suggesting that the CD27 CARs were able to specifically target tumor cells without affecting healthy immune cells [72].

A second receptor–ligand interaction used in CARs for GBM is the natural killer group 2 member D (NKG2D) receptor, which is expressed on natural killer (NK) and CD8^+^ T cells, and mediates their cytotoxic activity. In GBM and GBM stem cells, NKG2D ligands (NKG2DLs) are expressed at high levels, while most healthy tissues only exhibit low levels of expression [73, 74]. In one study, CAR T cells expressing the NKG2D extracellular region were produced to target NKG2DL-expressing GBM cells and GBM stem cells. Against both cell types, NKG2D CAR T cells had potent cytotoxic effects, and when the CAR T cells were administered to mice bearing NKG2DL-expressing GBM xenografts, the tumors diminished while the CAR T cells persisted for up to 45 days after infusion [75]. A second study utilized a separate NKG2D CAR construct and showed that 22% of mice bearing glioma achieved remission upon CAR T cell treatment. These mice were also able to survive a tumor rechallenge test without additional therapy, suggesting the long-term benefits of these CAR T cells. Based on prior studies indicating that irradiation can upregulate NKG2DLs on glioma cell surfaces, mice irradiated before infusion with CAR T cells showed prolonged survival and decreased tumor volume compared to treatment with NKG2D CAR T cells alone [76].

Chlorotoxin (CLTX), a 36-residue peptide isolated from scorpion venom, has also been incorporated into the extracellular recognition domain of CARs to target GBM [77]. This is based on the highly specific binding of CLTX to a membrane-associated protein, matrix metalloproteinase 2 (MMP2), which is overexpressed on glioma cells. The binding of CLTX to GBM cells has been shown to impair GBM migration and reduce its invasive capability [78]. In terms of reducing toxicities, CLTX itself is not toxic to normal tissues and does not bind to healthy tissues in the brain or elsewhere in the body [79, 80]. One study of CLTX CAR T cells indicated that these cells could target both GBM tumor cells and cancer stem cells, with over 80% of cells from surgical resection samples being able to bind to CLTX. Against GBM xenografts in mice, CLTX CAR T constructs were able to exert tumor control and lead to tumor eradication for over 170 days. *In vitro* rechallenge studies also indicated that CLTX CAR T cells were effective against multiple rounds of tumor challenge. No toxicities were observed in mouse models, supporting the favorable safety profile of CLTX CARs. This study also showed that CLTX binding to GBM cells was independent of expression of other tumor antigens, such as IL13Rα2, HER2/neu, and EGFR, meaning CTLX CAR T cells could target GBM cells with different antigen expression profiles [77]. When applied to patients, this may allow CTLX CAR T cells to be effective in a wider range of patients, as well as account for tumor heterogeneity within one patient.

## Expanding the function of glioblastoma-directed CAR T cells

Future CAR T cells might be most successful when constructed using multicistronic vectors, which contain 2A sequences that allow multiple genes to be incorporated into one cassette [46, 57]. These types of cassettes can expand the function of CAR T cells to allow them to express additional products. For example, multicistronic CAR constructs can target different antigens through BiTE co-expression, which have been shown in mouse models to effectively recognize EGFR on GBM cells when secreted by anti-EGFRvIII CAR T cells [46]. Increased clinical testing of these CARs against multiple antigens will be necessary to determine whether such constructs are viable in patients, but based on preclinical data, these constructs can address the obstacle of antigen escape in GBM.

A second application of multicistronic CAR vectors is to express genes for antibodies against immune inhibitors, which can increase CAR T cell persistence in the immunosuppressive TME. One mechanism of T cell inhibition is through the programmed cell death protein 1 (PD-1) pathway. PD-1, expressed on activated T cells, binds to PD-L1 on GBM cells; activation of the PD-1/PD-L1 axis hinders T cell responses [29, 30]. PD-1 blockade, where anti-PD-1 antibodies are used to prevent the PD-1/PD-L1 interaction, has been effective in previous clinical trials of GBM patients, both with and without CAR T cell therapy. In one clinical trial, pembrolizumab, an anti-PD-1 monoclonal antibody, was administered to GBM patients prior to surgical resection. This improved median overall survival by 6 months and induced greater T cell activation compared to patients who received adjuvant therapy, where pembrolizumab was administered after surgery [81]. A recent preclinical study combined EGFRvIII-specific CAR T cells with PD-1 blockade, as PD-L1 expression was detected on EGFRvIII^+^ GBM target cells and increased upon CAR T cell administration. Mouse models receiving EGFRvIII-specific CAR T cells and anti-PD-1 antibodies had increased survival and tumor clearance compared to mice receiving only CAR T cells [82]. Given the role of PD-1 blockade in improving T cell activity, co-expressing anti-PD-1 antibodies and CARs in T cells could lead to therapeutic benefit against GBM. While this has not been assessed specifically in GBM models, a proof-of-concept for this method has been established in studies targeting other malignancies [7]. One study modified CAR T cells targeting MUC16, an antigen found in ovarian cancers, to secrete scFvs against PD-1. In mouse models, treatment with these CAR T cells led to long-term T cell persistence and protection against tumor rechallenge [83].

Another method of stimulating CAR T cell activity and survival is to upregulate stimulatory cytokines in the TME. In a study using EGFRvIII-specific CAR T cells to treat glioma models, co-administration of local IL-12, a pro-inflammatory cytokine that has been shown to improve T cell cytotoxicity, led to tumor elimination and a decrease in exhaustion markers on the CAR T cells [84]. A second study transduced T cells with two vectors, one for an IL13Rα2-specific CAR and one encoding IL-15 to expand the function of the CAR T cells. When compared to CAR T cells that lacked the IL-15 construct, CAR T cells secreting IL-15 had delayed exhaustion and yielded longer progression-free survival in xenograft mouse models [85]. These results are congruent with previous data indicating that IL-15 promotes a stem-like T cell phenotype, which is associated with greater antitumor efficacy and proliferation [39, 86, 87]. Taken together, these studies provide support that the presence of stimulatory cytokines, whether through exogenous administration or transgenic expression, can benefit CAR T cell function against GBM. Armored CARs (Figs 1 and 3), where both the CAR construct and cytokine are encoded in one vector, have been generated for other solid tumors and produced promising results in terms of increasing CAR T cell persistence and exerting sustained antitumor effects [88–91].

Besides modifying CAR T cells to secrete cytokines, co-expressing cytokine receptors on CAR T cells can improve persistence and anti-tumor effects. Interleukin 8, a cytokine that can mediate metastatic spread, can also serve as a chemotactic signal for CD70-specific CAR T cells co-expressing either CXCR1 or CXCR2. In mouse GBM models, CAR T cells expressing CXCR1 or CXCR2 migrated to tumor sites more effectively than CAR T cells without these IL-8 receptors and were able to eliminate tumors almost entirely, leading to long-term survival [92]. Given that the CAR T cells in this study were intravenously infused, co-expressing cytokine receptors on CAR T cells may enhance trafficking to tumors by inducing chemotaxis. In another study, CAR T cells were transduced with a constitutively active IL-7 receptor, promoting downstream signaling in the absence of a ligand. Against mouse xenograft models, these T cells were able to eliminate tumors and prevent recurrence [93]. Thus, the ability to induce cytokine signaling through a constitutively active receptor can prolong CAR T cell viability without the need to co-express or administer the cytokine itself, reducing the risk of toxicities associated with systemic cytokine administration.

The rational engineering of cytokines can also provide opportunities to improve CAR T cell persistence and proliferation, a shortcoming in ACT against GBM (and, more generally, against solid tumors). The generation of orthogonal IL-2 receptor-ligand pairs [94, 95] could allow patient administration of relatively high cytokine doses in a safe manner. Engineered, orthogonal cytokines (also known as synthekines) would not be subject to the dose-limiting toxicities encountered in pleiotropic cytokines such as IL-2, as the stimulatory effects would be restricted to the infused CAR T cells. Additional work [96] has also generated hyper-stable, affinity enhanced IL-2 mimics. Crucially, these IL-2 neoleukines bind the IL-2R$\beta \gamma_{c}$ heterodimer but not the IL-2Rα (CD25) chain. Initial assessments [96] suggest that, by abrogating the interaction with CD25, these neoleukines can limit the proliferation of CD4^+^ FOXP3^+^ T_regs_ and display reduced toxicity compared to native IL-2.

While CAR T cells tend to have constitutive expression of their engineered receptors, synthetic Notch (synNotch) CAR T cells represent a method of regulating CAR expression, providing more control and specificity (Fig. 3). SynNotch CAR T cells express a synthetic receptor that activates a transcription factor upon recognition of a priming antigen, which then promotes expression of a CAR directed against a different killing antigen [97]. One recent study developed synNotch CARs using a priming receptor against EGFRvIII and a killing TanCAR against IL13Rα2 and EphA2. Against a mixture of EGFRvIII^+^ and EGFRvIII^-^ cells derived from a GBM cell line, killing occurred when just 10% of the target cells expressed EGFRvIII for priming and increased when the priming cell population was 50%, while no killing was observed when all cells were EGFRvIII^-^. In studies using mouse xenograft models of heterogeneous GBM, treatment with these synNotch cells led to complete tumor control and long-term remission, while treatment with constitutively expressed EGFRvIII CAR and TanCAR T cells resulted in tumor recurrence. Additional studies of the synNotch CAR T cells indicated that, compared to traditional CAR T cells, the synNotch mechanism favored lower expression of exhaustion markers and a stem central memory phenotype. Given that CAR T cells generated from stem-like T cell phenotypes have produced increased antitumor effects in other studies, these results indicate that synNotch CAR T cells are able to preserve this favorable state and specifically target GBM cells in a heterogeneous tumor [39, 98].

Engineering CAR T cells to overcome tumor heterogeneity and increase persistence are important features for addressing GBM, but the method of administering these cells is also a key consideration. In one clinical trial using CD19-targeted CAR T cells in B-cell leukemia patients, T cells trafficked to the CSF and, in patients with CNS leukemia, the CAR T cells were able to control and reduce the leukemia cell population. This provided early indications that CAR T cells administered systemically can traffic to the CNS, despite it being immune-privileged and protected by the blood–brain barrier [10, 99]. While CAR T cells targeting GBM have been delivered both through intravenous infusion and intracranial injection, recent preclinical studies suggest that intracranial injection may promote greater T cell infiltration into tumors [67, 76, 77]. Moreover, intraventricular delivery has been shown to provide increased tumor control, especially in cases where widespread lesions are present [36, 52, 53]. Although intracranial and intraventricular delivery are invasive from a clinical perspective and may not be practical for some patients, appropriate modifications to CAR T cell delivery methods could provide significant survival benefits.

## Closing remarks

Using standard therapies, GBM remains difficult to manage and treat, resulting in poor overall survival for most patients. Given the success of CAR T cell therapies in hematological cancers, there is untapped potential in ACT against solid malignancies such as GBM. Preclinical studies have characterized GBM antigens, including IL13Rα2 and EGFRvIII, as viable targets. These studies are supported by clinical trials, which have shown that CAR T cells can be safely delivered to patients to generate some antitumor benefits. At the same time, these trials have provided valuable insights on obstacles such as antigen escape and poor proliferation or persistence of CAR T cells. Recent experimental work has been instrumental in identifying alternative GBM antigens and novel CAR architectures to enhance CAR T cell survival and proliferation, with a view toward surmounting the immunosuppressive TME. Increased clinical work assessing the safety and efficacy of these new CAR designs can promote CAR T cells to become an established therapy that could be considered as part of the standard of care for GBM.

## Data Availability

Not applicable.

## Abbreviations

ACT: Adoptive cell transfer or therapy
BCMA: B-cell maturation antigen
BiTE: bispecific T cell engager
CAIX: Carbonic anhydrase IX
CAR: Chimeric antigen receptor
CLTX: Chlorotoxin
CNS: Central nervous system
CRS: Cytokine release syndrome
CSF: Cerebrospinal fluid
CSPG4: chondroitin sulfate proteoglycan 4
CTL: Cytotoxic T lymphocyte
DLT: Dose-limiting toxicity
EGFR: Epidermal growth factor receptor
EGFRvIII: EGFR variant III
EphA2: ephrin-A2
GBM: glioblastoma multiforme
HER2/neu: human epidermal growth factor receptor 2
IL: Interleukin
IL13Rα 2: Interleukin-13 receptor
MHC: Major histocompatibility complex
MMP2: Matrix metalloproteinase 2
MTD: Maximum tolerated dose
NKG2D: Natural killer group 2 member D
OS: Overall survival
OVA: Ovoalbumin
PD-1: Programmed cell death protein 1
PD-L1: Programmed death-ligand 1
PFS: Progression-free survival
scFv: Single-chain variable fragment
synNotch: Synthetic notch
TAA: Tumor-associated antigen
TanCAR: Tandem CAR T cell
T_CM_: Central memory T cell
TCR: T cell receptor
TME: Tumor microenvironment
TMZ: Temozolomide
TNF-α: Tumor necrosis factor-α
T_reg_: Regulatory T cell
TSA: Tumor-specific antigen
